# Editorial: Establishment of marker models for molecular typing of renal cell carcinoma

**DOI:** 10.3389/fonc.2023.1236980

**Published:** 2023-07-10

**Authors:** Aimin Jiang, Łukasz Zapała, Le Qu, Linhui Wang

**Affiliations:** ^1^ Department of Urology, Changhai Hospital, Naval Medical University, Shanghai, China; ^2^ Clinic of General, Oncological and Functional Urology, Medical University of Warsaw, Warsaw, Poland; ^3^ Department of Urology, Jinling Hospital, Affiliated Hospital of Medical School, Nanjing University, Nanjing, Jiangsu, China

**Keywords:** renal cancer (RC), tumor biomarkers, tumor prognosis, molecular subtype, multi-omics

According to the latest cancer statistics report, renal cell carcinoma (RCC) accounts for more than 400,000 new cancer cases and causes approximately 179,000 deaths worldwide (1, 2). Clear cell renal cell carcinoma (ccRCC) comprises approximately 75-80% of all cases of RCC, with the remaining percentage being represented by several subtypes of nonclear cell carcinoma (3). While curative treatment may be possible for patients with localized disease, others may present with metastatic or locally advanced disease. In some cases, patients with aggressive tumor biology may experience recurrence despite surgical resection. Given the variability in patient outcomes, accurate risk stratification is essential to identify patients who might benefit from more intensive initial treatment, closer monitoring, or adjuvant therapies. The advent of sophisticated multiomics techniques such as whole genome sequencing, combined with innovative bioinformatic tools, has enabled researchers to delve deep into tumor etiology and stratify patients based on characteristics associated with clinical outcomes. Based on the above concerns, there is an urgent need to identify novel biomarkers and risk models.

In this Research Topic, an overview of novel biomarkers and molecular subtyping of RCC is performed through 1 review and 15 original research papers by 119 authors, and these works facilitate our better understanding of cancer progression and heterogeneity to therapy response among RCC patients (Wang et al., Zheng et al., Pan et al. Lin et al., Zhang et al. Lin et al., Tao et al., Yu et al., Chen et al., Xia et al. Zhang et al., Chang et al., Teng et al., Zeng et al., Ding et al.).

Risk models based on transcriptome signatures could be better applied in clinical practice because of interpretability and accessibility. Wang et al. performed a comprehensive in silico combined with in-house validation analysis and divided ccRCC patients into CIN25-C1 and C2 subtypes based on 25 genes related to chromosomal instability. Patients with CIN25-C2 had a poor prognosis and increased proliferation, EMT, stemness and telomerase activity but were sensitive to sunitinib. There is great promise for the routine clinical application of CIN25-based ccRCC classification, as polymerase chain reaction (PCR) quantification appears to be sufficient. Lin et al. developed a reliable risk system based on ferroptosis and oxidative stress-associated genes and compared the differences at various levels, including clinical parameters, the immune microenvironment, and therapy resistance. They found that ccRCC patients with high risk scores had higher TMB levels and CD8^+^ T-cell infiltration degrees and preferable responsiveness to ICI therapy. Notably, a study from Pan et al. utilized the interferon regulatory family to construct a novel risk classifier for ccRCC with the application of a nonnegative matrix factorization algorithm, and they also applied the least absolute shrinkage and selection operator to develop a risk system to guide better risk stratification, which reached a superior performance than classical clinical parameters and the ClearCode34 model.

Accumulating evidence suggests that metabolic reprogramming, especially in fatty acid metabolism, is significantly correlated with tumorigenesis and progression in RCC. Ding et al. constructed an optimal nomogram consisting of the risk score of fatty acid metabolism-related genes and verified ten signatures involved in overall survival by immunohistochemical analyses, which also participated in uncontrolled pain in advanced RCC patients. Neutrophils are a type of abundant inflammatory cell present in the tumor microenvironment and could activate cancer cells and releasing modified DNA structures coated with cytoplasmic and granular proteins. A study from Teng et al. utilized neutrophil extracellular trap-related signatures to carry out a remodelling analysis and divided ccRCC patients into three distinctive subtypes with various activated states of metabolism and immune infiltration degrees, and four promising diagnostic genes, including *SLC27A2*, *SLC16A12*, *MAP7* and *SLC3A1*, were verified through RT−PCR.

Mitogen-activated protein kinase (*MAPK*) signaling is one of the most extensively studied pathways in tumor research. Zhang et al. constructed a risk score consisting of 14 *MAPK*-related genes using Lasso regression analysis and further proved that *MAPK* activation is correlated with various malignant behaviors of tumor cells, including but not limited to invasion, migration, apoptosis, and extracellular matrix degradation. Recent studies have found that the basement membrane, comprising fundamental components, displays crucial biological functions in the body by providing resistance against mechanical stress and determining tissue morphology and cancer progression. Tao et al. established a risk scoring system involving 16 basement membrane genes, which were related to metabolic and tumor-related signaling cascades. Studies have suggested the involvement of iron channels, especially potassium channels, in the proliferation and migration of various tumors by regulating T-cell function. Notably, Zeng et al. constructed a promising prognostic signature involving hypoxia and angiogenesis signatures based on potassium ion channel-related genes for ccRCC and finally validated the differential expression of four biomarkers related to potassium transport, including *ATP1A3*, *GNB3*, *GNB4* and *NSF*. The homeobox (*HOX*) family, encoding a conserved family of transcription factors in mammals, plays an indispensable role in organogenesis and development. A study from Zheng et al. reported an eight *HOX* gene-based risk model, and patients were divided into a lower risk group with a fragile type II IFN response and para-inflammation scores. Noninvasive surveillance approaches, especially liquid biopsy, are suitable for functioning as a repeatable and personalized snapshot among patients with high clinical stage scores. Zhang et al. carried out an integrative analysis consisting of transcriptomic and proteomic profiles and finally developed a risk score (containing *VSIG4*, *TFGBI* and *P4HB*) to predict the long-term prognosis of ccRCC patients with venous tumor thrombus.

Consistently, some promising diagnostic- and therapeutic-related targets specific for ccRCC were also investigated. Aided by systematic bioinformatic analysis and *in vitro* experiments, Xia et al. proved that T-cell immunoglobulin and the *ITIM* domain, or *TIGIT*, were highly expressed in tumor tissues and identified as crucial prognostic determinants. TIGIT might promote Treg cell infiltration, and patients with high expression of this signature might benefit from sunitinib treatment. In addition, two potential drugs (PD0325901 and selumetinib) targeting *TIGIT* were identified and verified by molecular docking. Chang et al. proved that the dysregulated expression level of one amino acid metabolism regulator, L-dopa decarboxylase (*DDC*), could trigger higher intratumoral heterogeneity and an immunosuppressive state in ccRCC *via* PI3k/Akt signaling after analysing multiomics profiles across four ccRCC datasets.

For advanced ccRCC patients, a second-line therapeutic strategy of axitinib is suitable to prolong progression-free survival after first-line therapies fail, while intra- and intertumoral heterogeneity could vary the therapy response rate. Lin et al. enrolled 44 advanced ccRCC patients and applied a combination of Cox and Lasso algorithms to construct a predictive model to predict the axitinib benefit rate. This model reached satisfactory performance, since the area under the curve values of 3-, 6-, and 12-month progression-free survival were 0.975, 0.909, and 0.911, respectively.

Genetic alterations, such as mutations and chromosomal copy number variations (CNVs), have emerged as an initial step towards genomic stratification in RCC. Tai et al. collected 55 patients with RCC across different regions in China with whole genome sequences and summarized the results as follows: In patients with ccRCC, the occurrence of mutations in *VHL, PBRM1, BAP1*, and *SERD2* reached 74%, 50%, 24%, and 18%, respectively. In contrast, among patients with nonclear ccRCC, the most frequently observed mutations were those in *FH* (29%), *MLH3* (24%), *ARID1A* (18%), *KMT2D* (18%), and *CREBBP* (18%). Previous genomic analysis of clinical samples of ccRCC unveiled a high incidence of *SETD2* mutations, which could expedite cancer progression through epigenetic regulation. Yu et al. provided a comprehensive summary of *SETD2* in ccRCC occurrence and progression, which suggested that hypermutated *SETD2* could be treated as a novel therapeutic target.

Although there are numerous prognostic biomarkers found in RCC that provide novel insights into diagnosis and therapy, their accuracy and utility remain to be further investigated and verified. The clinical utility and widespread application of specific risk models or biomarkers is hindered by numerous challenges, including resource limitations, complexity, the need for repeated outhouse validation, and ideally, evaluation across different prospective clinical trials. Nonetheless, our understanding of the biological mechanisms governing RCC initiation and progression continues to progress alongside the advances of new platforms for clinical application. In the future, it is possible that genomic or other profiling of each patient’s tumor might facilitate personalized medicine, enabling the administration of appropriate treatments to the right patients at the optimal time.

## Author contributions

LW and ŁZ supervised and conceived the topic. AJ and LW reviewed all articles on this Research Topic and wrote the original manuscript. LQ reviewed the manuscript. All authors approved the final version of this paper.
